# Protecting the Melatonin Rhythm through Circadian Healthy Light Exposure

**DOI:** 10.3390/ijms151223448

**Published:** 2014-12-17

**Authors:** Maria Angeles Bonmati-Carrion, Raquel Arguelles-Prieto, Maria Jose Martinez-Madrid, Russel Reiter, Ruediger Hardeland, Maria Angeles Rol, Juan Antonio Madrid

**Affiliations:** 1 Department of Physiology, Faculty of Biology, University of Murcia, Murcia 30100, Spain; E-Mails: mbc11365@um.es (M.A.B.-C.); raquel.arguelles@um.es (R.A.-P.); mj.martinezmadrid@um.es (M.J.M.-M.); jamadrid@um.es (J.A.M.); 2 Department of Cellular and Structural Biology, University of Texas Health Science Center, San Antonio, TX 78229, USA; E-Mail: REITER@uthscsa.edu; 3 Johann Friedrich Blumenbach Institute of Zoology and Anthropology, University of Göttingen, Göttingen 37073, Germany; E-Mail: rhardel@gwdg.de

**Keywords:** chronodisruption, circadian, light at night (LAN), melanopsin, melatonin

## Abstract

Currently, in developed countries, nights are excessively illuminated (light at night), whereas daytime is mainly spent indoors, and thus people are exposed to much lower light intensities than under natural conditions. In spite of the positive impact of artificial light, we pay a price for the easy access to light during the night: disorganization of our circadian system or chronodisruption (CD), including perturbations in melatonin rhythm. Epidemiological studies show that CD is associated with an increased incidence of diabetes, obesity, heart disease, cognitive and affective impairment, premature aging and some types of cancer. Knowledge of retinal photoreceptors and the discovery of melanopsin in some ganglion cells demonstrate that light intensity, timing and spectrum must be considered to keep the biological clock properly entrained. Importantly, not all wavelengths of light are equally chronodisrupting. Blue light, which is particularly beneficial during the daytime, seems to be more disruptive at night, and induces the strongest melatonin inhibition. Nocturnal blue light exposure is currently increasing, due to the proliferation of energy-efficient lighting (LEDs) and electronic devices. Thus, the development of lighting systems that preserve the melatonin rhythm could reduce the health risks induced by chronodisruption. This review addresses the state of the art regarding the crosstalk between light and the circadian system.

## 1. Evolution of Artificial Illumination

Light is a wave corresponding to a small part of the electromagnetic spectrum to which human eyes are responsive. The visible spectrum includes the range from 390 to 780 nanometers (nm), with a peak sensitivity at 555 nm [1].

Humans have been using artificial light for many years. The first evidence arises from attempts to control fire by *Australopithecus* 1.42 million years ago. After them, *Homo erectus* started to use fire in caves around 500,000 years ago [2,3]. In Greece, lamps made from pottery or bronze started to replace torches around 700 B.C. From that time until the nineteenth century, the most commonly used and advanced lighting tool was the wax candle [3]. However, these lighting methods produced lights of very low intensity and low color temperature, with negligible effects on the circadian system.

With the Industrial Revolution, lighting tools and technologies experienced a tremendous process of accelerated development and improvement after the discovery of the incandescence of an energized conductor by Humphrey Davy in 1801. This process culminated in the second half of the nineteenth century, with the practical use of the incandescent light bulb developed by Joseph Swan and Thomas Alva Edison [1]. Nowadays, the increased efficiency of electricity production and the reduction of the transport costs have contributed to the proliferation of electricity all around the world [1].

As a result, our homes and work places are currently illuminated and we no longer have nights of complete darkness. In fact, 2/3 of the population in the European Union regularly experience nights where the sky is brighter than under a full moon [4]. Moreover, since the 1960s, artificial lighting has tended to use increasingly higher intensity discharge lamps, which mainly consist of blue wavelengths [5,6] that affect the circadian system to a greater extent than any other. While daytime outdoor illuminance normally ranges from 2000 to 100,000 lux, indoor office lighting is usually significantly lower, with values around 500 lux [7]. Thus, humans have altered the natural light-dark cycle contrast, which may have serious pathophysiological repercussions [8].

## 2. The Functional Organization of the Human Circadian System

**Figure 1:**
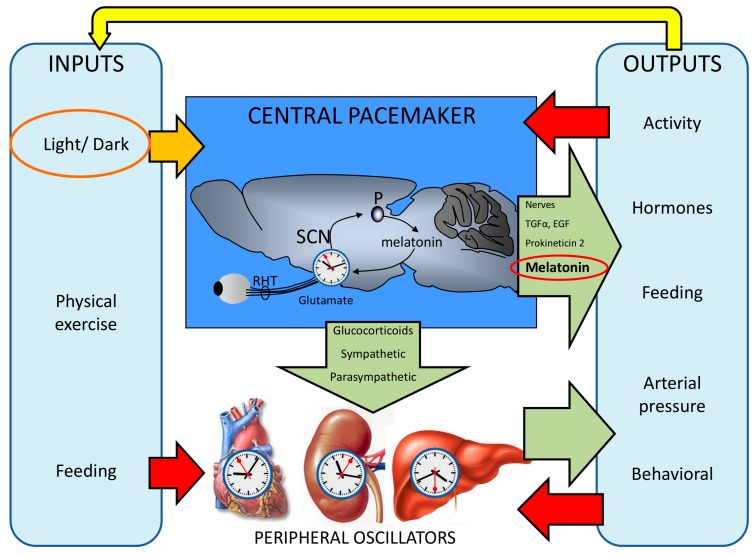
General overview of the functional organization of the circadian system in mammals. Inputs: environmental periodical cues can reset the phase of the central pacemaker so that the period and phase of circadian rhythms coincide with the timing of the external cues; Central pacemakers: the suprachiasmatic nuclei (SCN) is considered to be the major pacemaker of the circadian system, driving circadian rhythmicity in other brain areas and peripheral tissues by sending them neural and humoral signals (such as melatonin, secreted by the pineal gland (P)). The SCN receives light-dark cycle information through the retinohypothalamic tract (RHT). Peripheral oscillators: most peripheral tissues and organs contain circadian oscillators. Usually, they are under the control of the SCN; however, under some circumstances (e.g., restricted feeding, jet lag and shift work), they can desynchronize from the SCN; Outputs: central pacemakers and peripheral oscillators are responsible for the daily rhythmicity observed in most physiological and behavioral functions. Some of these overt rhythms (physical exercise, core temperature, sleep-wake cycle and feeding time), in turn, provide feedback, which can modify the function of the SCN and peripheral oscillators, (redrawn from [11]).

**Figure 2:**
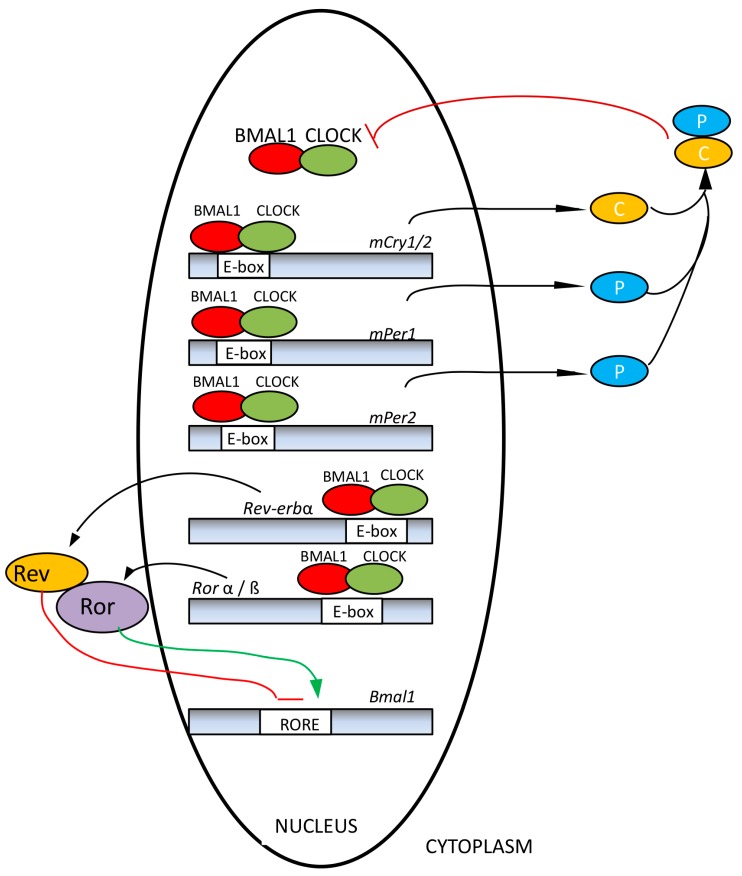
Molecular clock of mammals. Circadian locomotor output cycles kaput (CLOCK)/brain and muscle aryl hydrocarbon receptor nuclear translocator-like (BMAL1) heterodimers (red and green ovals) bind the DNA of clock target genes at E-boxes or E’-boxes and permit their transcription. The resulting period (PER) and cryptochrome (CRY) proteins (blue and yellow) dimerize in the cytoplasm and translocate to the nucleus where they inhibit CLOCK/BMAL1 proteins from initiating further transcription (redrawn from [16]).

The circadian timing system, a hierarchically organized network of structures responsible for generating circadian rhythms, is driven in mammals by a circadian pacemaker located in the suprachiasmatic nuclei (SCN) of the hypothalamus. It allows organisms to adjust their physiology by anticipating daily environmental changes, instead of merely responding to them in a reactive manner. Thus, under natural conditions, endogenous circadian rhythms are entrained to the 24 h light-dark cycle (for a review, see [9]). In humans, daily rhythms are observed in a variety of molecular, physiological and psychological processes, such as gene expression, body temperature, heart rate and melatonin production, as well as sleep, mood and higher cognitive functions (for a review, see [10]). The circadian system (CS) consists of [11,12] (Figure 1):

(a) Oscillatory machinery, including a central pacemaker, the SCN [13], and peripheral oscillators located in most tissues and cells [14]. Their rhythms are generated by a transcriptional-translational feedback loop between two groups of clock genes (positive and negative elements). Circadian locomotor output cycles kaput (*Clock*) and brain and muscle aryl hydrocarbon receptor nuclear translocator-like (*Bmal1*), acting as positive elements, are responsible for the synthesis of two transcription factors which, after heterodimerization, induce the expression of negative components of the molecular circadian clock, such as isoforms of Period (*Per 1*, *2*, *3*) and Cryptochrome (*Cry1* and *Cry2*) and a Nuclear receptor subfamily 1 (*Rev-Erbα*) [15,16] (Figure 2). An unknown clock gene, referred to as Chrono, has been recently added to this list. It seems to function as a transcriptional repressor of the negative feedback loop in the mammalian clock. Chrono binds to the regulatory region of clock genes, and its occupancy oscillates in a circadian manner [17]. Variants of the core oscillator alternately use not only orthologs (e.g., *Per 1*, *2*, *3*; *Cry1*, *2*, *Bmal1*, *2*), but also paralogs, such as NPAS2 (neuronal PAS domain protein 2), which can replace CLOCK. The variants can exist as oscillators acting in parallel in the same organ [18]. Moreover, the core oscillator system is associated with numerous, often tissue-specific accessory proteins that also undergo circadian cycling and additionally feed into the core oscillator. Among these, nicotinamide phosphoribosyltransferase (NAMPT) [19], peroxisome proliferator-activated receptor-γ (PPARγ) [20,21], sirtuin 1 (SIRT1) [22,23], AMP-activated protein kinase (AMPK) [24] and protein kinase Cα (PKCα) [25,26] are of particular importance, because they connect oscillators with metabolic sensing and mitochondrial function and are also controlled or modulated by melatonin [27]. As metabolic sensors, these accessory oscillator components are also relevant to health, especially with regard to metabolic syndrome and diabetes type 2, but also in the context of aging [28]. The connection between circadian oscillators and health maintenance extends to the prevention and suppression of cancer. Some core oscillator components, such as PER1 [29], PER2 [30,31,32] and BMAL1 [33,34], and the oscillatory output factor and modulator of Rev-erbα, deleted in breast cancer 1 (DBC1) [35], have been shown to act as tumor suppressors. These insights originated from the crucial finding that mice carrying a mutation in the *Per2* gene are cancer-prone [30]. Meanwhile, this conclusion has been supported by other data, including epigenetic knockdowns of core oscillator genes and oscillator dysfunction in cancer cells (summarized in references [18,28]).

(b) Input pathways carry information about the light-dark cycle to the central pacemaker. This pathway starts in a particular type of retinal ganglion cells containing melanopsin (which makes them intrinsically photosensitive). These cells are directly excited by blue light [36], and send the information to the SCN through the retinohypothalamic tract (RHT). In addition to their intrinsic photosignal, they receive rod and cone inputs [37,38]. Other synchronizers, such as feeding cycles, scheduled physical exercise and social activities, are also connected to the central pacemaker and peripheral oscillators, contributing to their synchronization [39]; however, only the light-dark cycle has been demonstrated to be a necessary and sufficient condition for circadian synchronization.

(c) Output pathways are responsible for the coordination of circadian rhythms between different functions and parts of the organism. These are the result of humoral mediators, such as prokineticin-2, which is able to generate the rhythm of locomotor activity [40], and neural outputs, such as the rhythmic change in the parasympathetic/sympathetic balance [41], or the pineal release of melatonin during darkness [42]. This ubiquitous molecule is present in all biological domains, and it has been adopted during evolution as a “darkness molecule”. Its original antioxidant function, as well as its photosensitivity, caused it to be consumed during the day time (reducing oxidized molecules), thus peaking during the night [43]. In mammals, melatonin is produced by the pineal gland at night, and its secretion is inhibited most efficiently by light at ~460–480 nm. It is important to mention that these outputs can also act as inputs in a feed-back loop.

Neuroanatomical and functional studies point to the existence of nervous pathways between the SCN and heart, pancreas, liver, thyroid and pineal gland [41,44,45,46]. Using such means of communication, the SCN csan activate or silence different tissues, depending on their function at different times of the day. Thus, the circadian system functions as an orchestra, in which the SCN acts as the conductor and the peripheral oscillators are the different instrumental groups.

The cycle of sunrise and sunset has provided a reliable time cue for many thousands of years, until recently when modern life and the “24-hour society” intensified exposure to artificial lighting environments, both during the day and at night, as people engage in shift work and leisure time is displaced towards the nighttime hours. Thus, it is important to find a way to illuminate the night that permits the circadian entrainment and respects the melatonin rhythm. Reducing the blue component of nocturnal light could prevent light-induced disruption of the circadian system and provide an attractive means of reducing the health risks induced by LAN.

## 3. Influence of Unfavorable Illumination on Human Health

Considering that contemporary humans spend most of their time indoors [47,48,49,50], with very low levels of natural light and with artificial light sources during both day and night [51], the classical conception of light as a mere input to the circadian system should be questioned. Light exposure is voluntarily and also unconsciously manipulated to match rest-activity rhythms, as well as work and leisure activities. The rhythm of light exposure should, therefore, be considered simultaneously as an input, and, in some aspects, a result of circadian system function, which in turn provides feedback to the suprachiasmatic nuclei (SCN). Unlike our ancestors, who lived in natural environments, the most recent generations of people residing in developed countries have self-selected their light-dark cycle. The main differences between these two lifestyles with regard to light exposure are a progressive overall decrease in light intensity and regularity; a modification in light timing, with delayed and reduced exposure during the day and increased light at night; and a shift in the light spectrum towards artificial light sources with a strong blue component. These changes in light input are hypothesized to be the reason why a large proportion of people suffer from some degree of chronodisruption in modern society [8,52,53,54].

There are some situations in which individuals are particularly exposed to a chronodisruptive illumination, with significant effects on human health. Among them are changes in light exposure based on different latitudes and special situations, such as shift work or jet lag (including social jet lag). This section is dedicated to a review of their effects on the circadian system.

### 3.1. Latitudinal Influence on Light-Dark Cycle

Regarding the differential effects of ambient temperature and light exposure depending on latitude, it is obvious that living in the polar regions entails an ability to adapt to extremes of cold, day length, and in some circumstances, isolation for long periods of time [55].

Recently, it was again emphasized that the extreme light conditions of the polar regions offer promising leads for epidemiological studies into light-associated diseases, including cancers, especially considering that there is a low incidence of hormone-dependent cancers [56,57] such as uterine, ovarian and prostate cancer, which are indeed rare in the Arctic as compared to populations from lower latitudes [57]. Although we cannot discard other factors, such as diet, genetics, *etc.*, these unusual patterns of cancer in residents of the Arctic regions would be compatible with an enhanced overall annual amount of melatonin, a compound shown to possess oncostatic and, presumably, also cancer-preventive properties [58,59,60,61,62]. Therefore, annual melatonin patterns, together with analytic epidemiologic data, might provide important clues to hormone-dependent carcinogenesis [57].

Existing evidence suggests that the circadian system is disturbed during the polar winter, largely due to insufficient bright light [55]. This perception was reinforced when Seasonal Affective Disorder (SAD) was classified as an illness in 1984. The disorder, which is characterized by recurrent episodes of major depression with a distinct seasonal pattern, was attributed to long winter nights and could be successfully treated with extra bright light, which mimics a long summer day. The light therapy is generally well tolerated, with most patients experiencing clinical improvement after one or two weeks of treatment; it can even be prophylactically prescribed before autumn. Also, to prevent relapse, it is appropriate to continue with light therapy throughout the winter, until the spontaneous remission of symptoms in the spring or summer [63].

### 3.2. Shift Work

Approximately 15%–20% of workers in Europe and the US participate in shift work, including work at night [64]. The prevalence exceeds 30% in the manufacturing, mining, transport, health care, communications and hospitality sectors [65].

Night shifts involve being in the presence of artificial light at night. This disturbance in the “natural” daily light/dark cycle is responsible for an impaired melatonin rhythm [66], as well as a disruption of the circadian oscillator [67]. Both factors have been shown to be involved in several metabolic and endocrine disorders, as described below.

Epidemiological studies of shift-workers have demonstrated increased risks of breast [68,69,70], prostate [71], colorectal [72] and endometrial cancers [73]. These epidemiological data have been explained by a disruption of the circadian oscillator, as well as by the exposure to artificial light at night and the subsequent decrease in melatonin levels. Since the circadian oscillator is involved in the major cellular pathways of cell division, its disruption may be linked to disturbances in cell cycle control [74], which has been associated with cancer and acceleration of malignant growth, possibly as a result of the interruption of DNA damage check-points [74]. Moreover, several studies indicate that exposure to LAN affects the transcription level of a substantial number of genes that are associated with cell cycle progression, cell proliferation and tumorigenesis [75].

LAN has been shown to disrupt the circadian rhythm of hormone production, as in the case of leptin [76], and particularly melatonin, which has been linked to an increase in cancer risk in shift-workers [54,77,78]. This disruption of the hormones production rhythm is additionally aggravated by unconventionally-timed synchronizing cues via food intake [79,80] and other activities at unusual times, which result in inappropriate and confusing entrainment information.

It should also be noted that night shift workers have the added obstacle of switching back and forth between weekday or working days and the weekend, or schedules with days off; this results in an additional increase in circadian desynchronization [81]. Once again, this certainly leads to melatonin disturbances and chronodisruption.

### 3.3. Jet Lag/Social Jet Lag

Jet Lag Disorder falls into the category of Circadian Rhythm Sleep Disorders (CRSD) in the International Classification of Sleep Disorders (ICSD-2) [82]. It is generated by rapid travel across multiple time zones, a change too drastic to allow the circadian system to adapt smoothly [83,84]. The most common jet lag symptoms include sleep impairment, rhythm desynchronization, anxiety and depressed mood, gastrointestinal and cardiovascular complaints, dizziness and menstrual irregularity in women [85].

The organization of work schedules, and sometimes also leisure activities, interferes with individual sleep preferences. In late chronotypes, the constraints of early work schedules lead to an increasing sleep debt over the week that is compensated on weekends. The fact that many individuals shift their sleep and activity times by several hours between the work week and the weekend (or other free days) induces “social jet lag”, which is comparable to jet lag [86]. To worsen the problem, the internet, email, video games and television also contribute to later bed times, as well as to expose to light-emitting diode (LED) screens, which have been shown to suppress melatonin secretion [87,88] and disrupt sleep [89]. Light exposure after sunset causes delayed shifts in the clock and a later onset of melatonin secretion, which could contribute, at least in part, to reducing the hours children sleep by about 1.2 h on school nights as compared to the average hours they slept a century ago [90]. Among adolescents, 25% of high school students in Japan [91], 16.1% in China [92], 22.8% in the USA [93] and 9.9% in Spain [94] suffer from insomnia. All of this presumably results from disturbed habits and irregular lifestyles. The expanding use of leisure technology seems to have substantially contributed to this sleep deficiency [95].

Although the above-mentioned lifestyle habits may not be easily changed, the use of appropriately timed light with suitable spectral properties should be promising, at least in the treatment of jet lag. Despite the awareness of the predominant role of light in regulating circadian rhythms, surprisingly few detailed studies have systematically documented its efficiency in reducing the symptoms of jet lag. A study by Eastman and colleagues (2005) compared sleep advances of one and two hours, combined with three hours of intermittent bright light treatment (5000 lux 30 min on, 30 min off) and concluded that the one hour sleep schedule advance was as effective as the two hour advance schedule [96]. This study indicates that a pre-flight light treatment in the morning can be beneficial. However, exercise has also been shown to have a potent effect in resynchronizing rhythms and reducing jet lag symptoms [85]. Another recommendation related to the symptoms of jet lag is to avoid sunlight at certain times when traveling through more than six time zones (e.g., [97]; reviewed in [98]).

In addition, melatonin is effective in preventing or reducing jet lag [99,100,101,102,103], and there is no evidence of any meaningful side effects. Melatonin is commonly used by adult travellers flying across five or more time zones, and sometimes even when crossing only 2–4 time zones, especially in an easterly direction [104].

A special condition that deserves attention is hospitalization. Intensive care units constitute another chronodisruptive situation in which patients spend their time in noisy and illuminated environments during the entire day and night [105,106] with a high number of care interactions per night and patient (for more details, see [107]). These unnatural ambient conditions have been shown to influence the appearance of sleep problems, melatonin suppression (for a review, see [107]) and are closely related to delirium [108]. It has been recommended to reduce noise and light levels during the night, so that sleep is less disturbed [106], although attempts at correcting the melatonin level by means of a light/dark cycle have been unsuccessful [109].

### 3.4. Chronodisruption

The above-mentioned conditions are the cause of circadian disruption or chronodisruption (CD). This term refers to a prolonged impairment of physiological, behavioral and biochemical rhythms within the organism [8]. The impairment can be detected by a loss of rhythmicity, increase in phase instability, extreme phase advances or delays or the appearance of internal desynchronization among the rhythms of different variables within a subject [11]. However, from an operational point of view, CD can be defined as the split of the physiological nexus between internal and external times [110]. Recently, some attempts have been made to develop objective indexes to determine circadian disruption. The circadian function index (CFI) described by Ortiz-Tudela *et al.*, 2010 [111] provides a quantitative score of a circadian rhythm based on three parameters: interdaily stability, intradaily variability and relative amplitude. Using CFI, specific populations, such as cancer patients [112], newborns [113] or individuals with metabolic syndrome [114], can be classified according to their circadian system status. Similarly, Erren & Reiter (2013) [110] proposed a quantitative characterization of CD suitable for epidemiological studies, based on the overlapping of internal time (determined by the mid sleep time) and the external time (determined by the interaction between the sunlight time and the social time imposed by working schedules). Although theoretically good circadian synchronization between internal and social time could be also obtained in people living regularly with their activity phase during nighttime, in the real world, late chronotypes and permanent nocturnal workers show a higher incidence of circadian system impairment and pathologies associated with chronodisruption than people living in synchrony with natural sunlight. The repeated disruption of the circadian system in humans [54,115] has been associated with several health impairments, such as metabolic syndrome [11,116], cardiovascular diseases [117], cognitive impairments [118] and a higher incidence of breast cancer [68,69] among others. Inadequate timing, spectrum and intensity of retinal light input produced by nocturnal activities and sleep during daylight is a key factor to explain the incidence of CD, since it not only induces instability in the master pacemaker, it also reduces melatonin synthesis [8].

## 4. Light Input Pathways

### 4.1. Intrinsically Photosensible Retinal Ganglion Cells (ipRGC)

Retinal ganglion cells transmit light information through the optic nerve. Their axons reach, among other targets, the dorsal lateral geniculate nucleus (dLGN) and other regions involved in conventional image vision. However, some RGC axons also send light information to brain centers for “non-image”-related visual functions, such as circadian photoentrainment [119]. These cells are the intrinsically photosensitive retinal ganglion cells (ipRGC).

**Figure 3:**
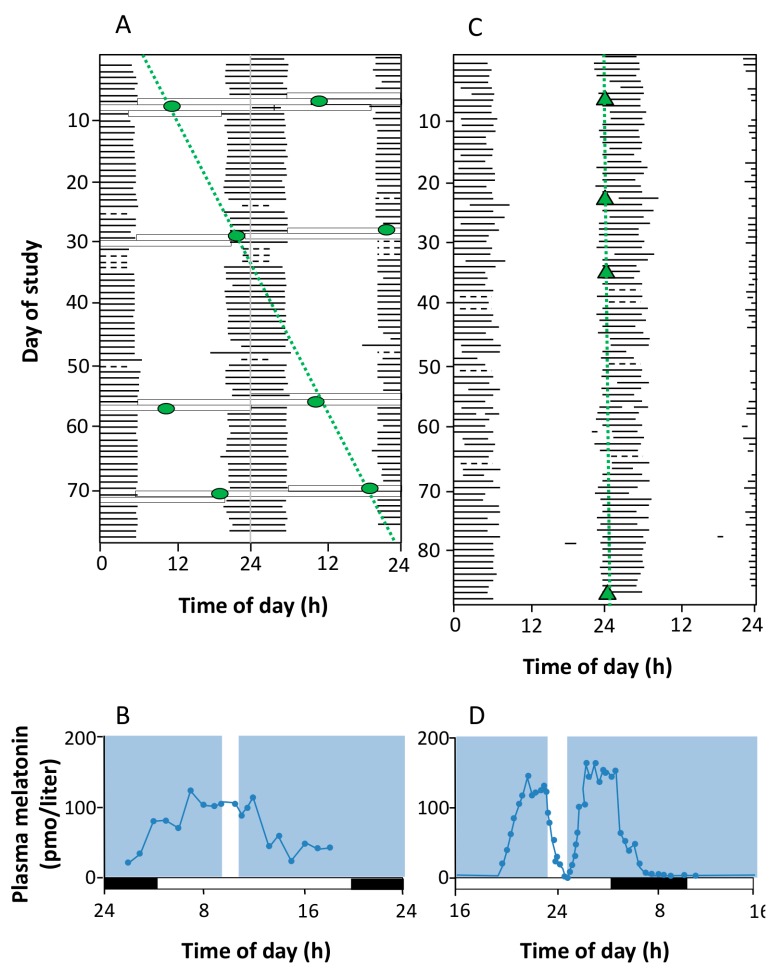
Absence and presence of circadian photoreception in two totally blind subjects. **A** and **B** correspond to the sleep-wake pattern and the results of melatonin suppression test in a 70-year old blind patient with congenital glaucoma who reported no conscious light perception and whose electroretinogram (ERG) and visually evoked potential (VEP) responses were not detectable. In (**A**), the sleep-wake pattern is double-plotted according to time of day (abscissa) and study day (ordinate). It is evident that the subject’s circadian system was not entrained to the light-dark cycle, and the core body temperature rhythm (circle) exhibited a non-24-h period; (**B**) shows the null effect of light (white bar) on melatonin secretion; **C** and **D** correspond to a 21-year-old woman with Leber’s congenital amaurosis, a type of retinal dystrophy. The ERG was undetectable, but an abnormal VEP was recorded. As represented in **C**, her circadian system was normally entrained (24-h period) and melatonin secretion was suppressed when she was exposed to light. Both results indicated that this patient, despite her lack of conscious light perception, preserved the retina-SCN-pineal pathway (reproduced from [122]).

Until recently, only two types of photoreceptors were known to exist in mammals: rods and cones. However, in 1923, it was suggested that a third photoreceptor must be involved in the pupillary light reflex and other non-visual light responses, since the eliciting light stimulus wavelength did not match those known for rods and cones [120]. In 1980, it was demonstrated that light continued to modulate dopamine levels in the retina with degenerated cones and rods [121]. In the 1990s, Czeisler *et al*. (1995) [122] reported cases of circadian system entrainment and melatonin secretion inhibition in response to light in blind people with no conscious light perception resulting from the severe loss of rods and cones (Figure 3).

Years later, studies on mice in which rods and cones were completely absent demonstrated that they still responded to light (at a wavelength of around 480 nm) with melatonin suppression [123], phase shift [124] and pupillary constriction [125]. Although these results could be strongly influenced by developmental rewiring, since the mice were obtained from knockout animals, these findings suggested that there must be an extra photoreceptor apart from rods and cones. In parallel, Provencio *et al*. (1998) [126] described a new photopigment in the skin of *Xenopus laevis*, which they called melanopsin. Two years later, the same authors found this pigment in a group of RGCs [127], and just one year later, Berson *et al*. (2002) [36] demonstrated that a group of RGC projected to SCN and responded to light even after blocking rods and cones. It was subsequently demonstrated that the “unknown” RGCs, which projected to the SCN and contained melanopsin, were one and the same [128].

In adult mammals, melanopsin appears to be expressed only in ipRGCs [119]. These ipRGCs show sparse, irregular and far-ranging dendrites, which also present prominent varicosities with an enrichment of mitochondria [129]. In the macaque, it has been shown that ipRGCs constitute 0.2% of the total RGC population, and some are stratified in the OFF-sublamina of the inner plexiform layer, some in the ON-sublamina, and some are bistratified. These different locations constitute three different subtypes [37].

Melanopsin belongs to the rhabdomeric group of visual pigments, which are predominantly found in invertebrates [130,131]. As a main characteristic, it has an unusual tyrosine residue in the counterion position for the Schiff-base linkage between the chromophore and the opsin protein [126,127]. It also presents a long and glycosylated [126] intracellular tail with potential phosphorylation sites [132]. In biochemically purified melanopsin from native ipRGCs, the absorption spectrum has been found to peak near 480 nm [133].

Regarding phototransduction, there are some differences between the classical process of rods and cones and that of the ipRGCs. In rods and cones, phototransduction involves a bleachable photopigment (rhodopsin), a transducin (G_T_) and a phosphodiesterase (PDE). In darkness, cGMP keeps the nonselective cation cyclic-nucleotide-gated (CNG) channels open. When light reaches the photoreceptor, the cromophore 11-cis-retinal converts into all-trans-retinal. This promotes a conformational change in the opsin, the consequence of which is the activation of PDE, which hydrolyzes cGMP, allowing the channel to close, and producing membrane hyperpolarization [130,131]. In ipRGCs, although the first step is also the transformation of 11-cis-retinal into all-trans-retinal, the coupled protein is G_Q_ and its activation promotes phospholipase C activation [130,131]. Although the subsequent steps in the activation cascade are still unclear, proteinkinase C (PKC) seems to be implied, triggering Ca^2+^ ion influx via transient receptor potential channels (TRPCs), eventually producing depolarization [36,37,131]. Another difference between melanopsin and the traditional photopigments is its bistability. While rhodopsin requires a complex machinery in the retinal pigment epithelium (RPE) to recover the 11-cis chromophore conformation, melanopsin (like the invertebrate photopigments) is able to recover the active conformation simply by absorbing a second photon from a longer wavelength [134].

**Figure 4:**
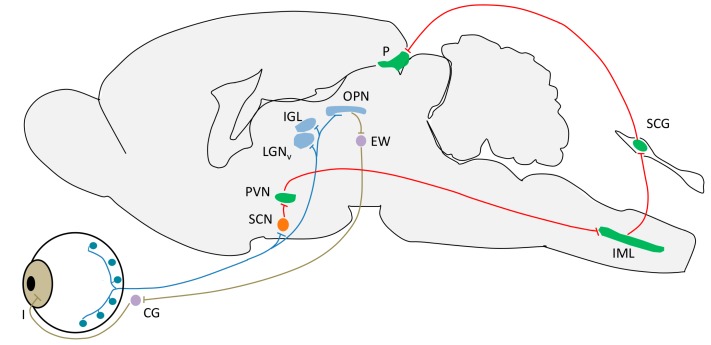
Schematic view of brain regions and circuits inervated by intrinsically photosensitive retinal ganglion cells (ipRGCs). The location of their somas, axons and main targets are represented in blue. Projections of ipRGCs to the SCN (orange) allow photic entrainment of the circadian clock. The red pathway with green nuclei represents a polysynaptic circuit originating in the SCN, which photically regulates melatonin release by the pineal gland (P) through sympathetic innervation. Synaptic links in this pathway include the paraventricular nucleus (PVN) of the hypothalamus, the intermediolateral nucleus (IML) of the spinal cord and the superior cervical ganglion (SCG). The olivary pretectal nucleus (OPN) is another direct target of ipRGCs, and is a crucial link in the circuitry underlying the pupillary light reflex, shown in brown (fibers) and purple (nuclei). Synapses in this parasympathetic circuit are found at the Edinger-Westphal nucleus (EW), the ciliary ganglion (CG) and the iris muscles (I). Other targets of the ipRGCs include two components of the lateral geniculate nucleus of the thalamus, the ventral division (LGNv) and the intergeniculate leaflet (IGL) (reproduced from [138]).

The axons of ipRGCs project to several regions in the brain. The most notable are the SCN (the master circadian pacemaker) through the RHT, the intergeniculate leaflet (IGL, a center for circadian entrainment), the olivary pretectal nucleus (OPN, a control center for the pupillary light reflex), the ventral subparaventricular zone (vSPZ, implicated in “negative masking” or acute arrest of locomotor activity by light in nocturnal animals), and the ventrolateral preoptic nucleus (VLPO, a control center for sleep). There are other projections whose functions are not so clear, including the lateral habenula and amygdala [128,135,136,137,138] (Figure 4). Furthermore, these ipRGCs receive rod and cone inputs [37,38], constituting the extrinsic input pathway.

Although ipRGCs are not considered an intrinsic component of the circadian clock itself, they are involved in some essential processes related to it:

1. Circadian photoentrainment. It is known that the circadian rhythm of “blind” patients that have lost image vision owing to rod/cone degeneration, but not ipRGCs, can still be photoentrained (and therefore can recover from jet lag, for example) [139,140]. Studies in melanopsin-null mice given a light pulse at the beginning of their rest or active period demonstrate the importance of ipRGCs in this process. The phase-shift induced was lower in these mice as compared to the wild-type, indicating that the contribution of rods and cones to this process is no higher than 50% [141].
2. Negative masking. Light reduces locomotion in mice and other nocturnal mammals. This effect is called “negative masking” and it has been demonstrated that melanopsin is required for a maximal and sustained response. Low intensity light promotes a “positive masking” (increase of locomotion), and it has been demonstrated that while rods and cones drive positive masking at very low intensities (this could imply that the image-forming system helps guide locomotion, or it may simply be due to the rod to ipRGC connections), these photoreceptors, together with ipRGCs, drive negative masking at higher light intensities [142].
3. Sleep regulation. In nocturnal rodents, a pulse of light during the dark period induces sleep and c-fos expression in the VLPO nucleus [143], a sleep promoting brain area, while a pulse of darkness administered during the light period can induce awakening [143,144]. Melanopsin-null mice lack these effects and they show perturbations in sleep homeostasis [143]. These findings can be applied to diurnal organisms, but light would promote awakening and darkness would facilitate sleep [145].
4. Suppression of pineal melatonin. In rodless/coneless mice, melatonin suppression is complete under high light intensity [146,147], a process which requires melanopsin. Moreover, some people who suffer from blindness as the result of a severe loss of rods and cones also show this melatonin suppression, with a spectral sensitivity consistent with melanopsin signaling [148,149,150].
5. Pupillary light reflex. The pupillary light reflex (PLR) allows reducing the rod and cone saturation by light, and improves resolution by increasing the depth of field. Because of its immediacy, the PLR is the most readily quantifiable behavior driven by ipRGCs. It is known that ipRGCs are necessary for reaching the maximal pupil constriction and for sustained constriction for long durations (perhaps to compensate for light adaptation in the rods and cones) [151]. In rodless/coneless mice, the PLR begins only in bright light, but it is driven until completion [146,152,153,154].

#### 4.1.1. Why Is PLR a Reliable Method to Assess Photoreceptor Contribution?

**Figure 5:**
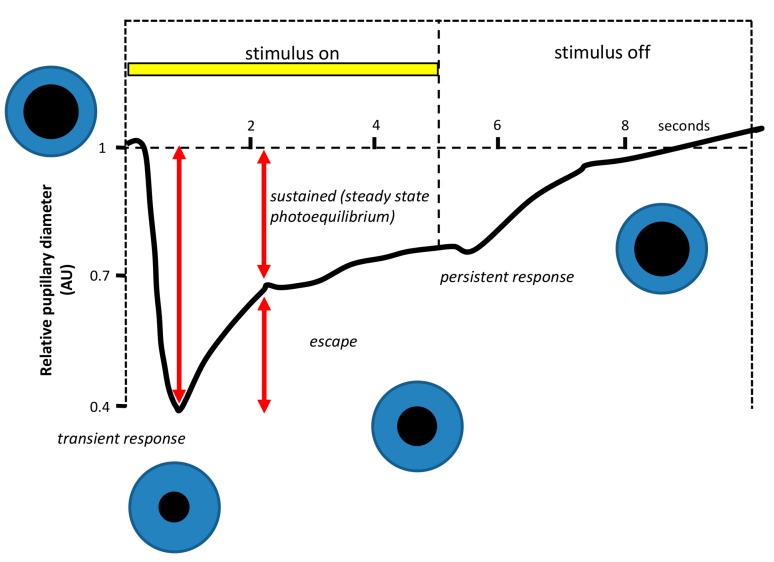
Example of a pupillographic recording in response to a 5-s bright white light stimulus in a normal human subject. The response waveform during the constriction phase has two components. When the light is turned ON, there is transient phase characterized by a short-latency, high-velocity maximal change in pupil size. Thereafter, the pupil partly redilates, or escapes, to a state of partial pupil constriction that represents the sustained phase of the pupil light reflex. When the light stimulus ends, the pupil starts to recover its original size after a period (which does not always occur) in which some degree of contraction persists after the light stimulus (modified from [155]).

**Figure 6:**
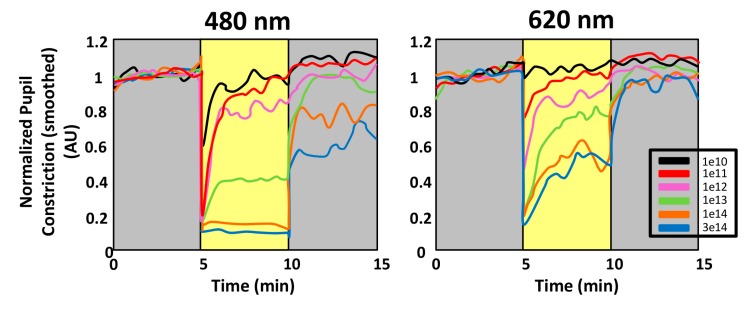
Spectral responses of the pupillary light reflex (PLR). Comparison of the PLR to 480 and 620 nm monochromatic long-duration (5 min) stimulations at 6 different irradiances in a single subject. After the initial and rapid pupil constriction, the steady state equilibrium and the persistent responses are present in all except the lowest irradiances, thus depending on wavelength and light intensity. The amplitude of the steady state equilibrium response is rapidly attained and particularly robust at 480 nm for the highest irradiances used. The persistent responses are also greater at 480 nm, as compared to 620 nm at equivalent irradiances. Note that for the higher irradiances, the pupil has not yet returned to the baseline within 5 min after extinction of the stimulus (reproduced from [134]).

Figure 5 shows a typical pupillary light response, consisting of two components. When the light stimulus is turned ON, a high-velocity pupil constriction ensues until it reaches a minimum pupil size (maximal constriction amplitude). This early transient response is followed by a pupillary redilation (escape) to a more sustained state of partial pupil constriction, which continues until the end of the light stimulus [155]. Studies in primates and humans suggest that the early transient pupil constriction under photopic conditions is a predominantly cone-driven response, while the sustained pupil constriction represents a summation of the adapted cone response and the steady-state intrinsic retinal ganglion cell activation [120,156] (Figure 6). Thus, the analysis of the transient and sustained pupillary response to light stimulus of different wavelengths, intensities and durations may be a good way to independently assess rod and cone function and the intrinsic activation of ipRGCs [155]. This could have clinical importance in order to differentiate normal from diseased eyes and pathologies of the rods and cones from those of the retinal ganglion cells or of the optic nerve. Moreover, PLR also represents a quick and easy tool to evaluate the effect of different light sources on ipRGCs, and thus on the SCN, through the activation of the first point of the entry of light in the circadian system.

#### 4.1.2. Circadian Rhythmicity in the Retina: Its Role in the Circadian System

In addition to the light-driven daily signals, retinal circadian signaling can also occur without light-dark stimulation, and there are ocular rhythms that do not depend on the presence of the SCN [157]. Interestingly, ipRGCs have been shown to express clock genes [158] and their light responses are modulated in a circadian rhythm and by dopamine [159,160], suggesting the possibility that the retina-driven rhythms observed in the SCN are originated in the ipRGCs themselves, or in retinal neurons with synaptic input to the melanopsin-expressing ipRGCs.

Thus, the mammalian retina contains a complete circadian clock system with biochemical machinery that generates temperature-compensated 24-hour oscillations (molecular clock) [158], an input pathway through which light synchronizes the rhythm of the retinal clock to the environmental light-dark cycle, and neurochemical output pathways that transmit the clock’s signal throughout the retina and into the rest of the brain. By controlling gene expression, synaptic communication and metabolism, the retinal circadian clock drives retinal circuits and their functioning reconfiguration according to the time of the day, thus allowing the anticipation of the normal cycle of photopic and scotopic visual conditions that alternate with the cycling of solar day and night. The molecular basis of the retinal circadian clock is, in principle, the same as that found in the SCN and other peripheral tissues [161].

The processes regulated in a circadian manner comprise melatonin formation and release ([162,163,164], dopamine (DA) synthesis [165,166,167], γ-aminobutyric acid (GABA) turnover rate and release [168], electroretinogram (ERG) b-wave amplitude [169,170], rod disk shedding [171], and UV opsin and rhodopsin gene expression [172]. Furthermore, the susceptibility of photoreceptors to degeneration from light damage [173,174], photoreceptor survival in animal models of retinal degeneration [175], and photoreceptor and retinal ganglion cell viability during aging [176] are also influenced by this retinal clock. But it remains unknown how this clock is configured.

Clock gene mRNAs and proteins have been mainly found to be concentrated in the inner nuclear layer of the retina [158,177,178,179], especially in dopaminergic amacrine cells [179,180]. Since DA is secreted with a circadian rhythm (with higher levels during the daytime, secreted in response to light) these cells could be the loci of the retinal circadian clock [179,180], although melatonin is required for the circadian regulation of DA release (and not *vice versa*) [181,182]. Melatonin has been found to be synthesized by photoreceptors [183,184] in a circadian manner (under the influence of *Bmal1* and *Clock* genes), promoting dark-adaptive effects. This suggests that rods and cones would also constitute putative clock cells.

Thus, dopamine and melatonin are key elements of the ocular circadian system, involved in many aspects of retinal physiology and pathology [185], and they allow the eye to enhance light-adapted cone-mediated visual function during the day and dark-adapted rod-mediated visual signaling at night (reviewed in: [185,186]). GABA, whose function on the retinal clock is less clear, is secreted mainly at night by horizontal and amacrine cells [168], and it is the principal fast inhibitory neurotransmitter in the retina, suggesting that its signal would reinforce the night phase [161].

ipRGCs also express clock genes and show daily rhythms in the expression of the melanopsin pigment gene, influenced by light and dopamine [187,188,189]. This also suggests that there might be clock neurons generating endogenous circadian rhythms, which are involved in the generation and entrainment of retinal circadian rhythms. Indeed, there are daily rhythms in the responsiveness of ipRGCs to light [159], and in the amplitude of the pupillary light response driven by these cells [190].

The dopaminergic amacrine cells of the INL receive their circadian light input from rods and cones through bipolar cells, and from ipRGCs through retrograde intra-retinal transmission [191,192]. Photoreceptor cells, which synthesize retinal melatonin, seem to receive an additional photic input indirectly through feedback from dopaminergic amacrine cells. Thus, it has been speculated that the retrograde drive of the ipRGCs on dopamine cells is a key factor in circadian entrainment of the retinal clock [192]. The intriguing consequence might be that ipRGCs would serve the entrainment of both the retinal and brain clocks, ensuring synchronization of these two neural oscillators [191].

### 4.2. Daytime Light Exposure Effects Mediated by Skin

The skin is exposed to solar radiation during day-time, which triggers neuroendocrine activities [193,194,195] and produces molecules that serve as biochemical readings of the circadian rhythm on the systemic level, including melatonin and serotonin, glucocorticoids [196,197] and CRH and POMC peptides [198].

## 5. Output Pathways: Melatonin

The SCN steers numerous rhythmic functions via a number of neuronal output pathways to hypothalamic and thalamic nuclei and structures. Among these, the paraventricular nucleus of the hypothalamus (PVN) is the first relay station towards the pineal gland. This neuronal pathway is extended even further, via the intermediolateral column of the upper thoracic cord to the superior cervical ganglion, from which postganglionic sympathetic fibers innervate the pineal and control melatonin synthesis through β- and α_1_-adrenergic stimulation. Further details and modulation by other neurotransmitters (PACAP, VIP, NPY and glutamate) have been summarized elsewhere [199]. However, other efferences from the SCN also exist, e.g., to the preoptic area, lateral septum, bed nucleus of stria terminalis, subventricular zone, arcuate nucleus, paraventricular nucleus of the thalamus and, perhaps, the amygdala, habenula and intergeniculate leaflet [200]. In functional terms, the importance of the pathway via the PVN to rhythmic functions lies beyond pineal activity, in particular, because it controls both parasympathetic and sympathetic projections and, additionally, influences the glucocorticoid rhythm, which is otherwise generated by an autonomous adrenocortical clock. However, glucocorticoid secretion can, in turn, be modulated by melatonin [201,202]. The absence of robust adrenocortical rhythmicity in melatonin-deficient C57BL mice [203] may be taken as an additional indication of the need for melatonin in the glucocorticoid rhythm, but this would need direct experimental support. Another SCN output of relevance to rhythmic organization and chronodisruption controls the hypothalamic sleep switch, a structure generating on-off responses on the basis of mutual inhibition. It alternately activates either wake-related downstream neuronal pathways that involve the locus coeruleus, dorsal raphe nucleus and tuberomammillary nucleus, or sleep-related pathways via the ventrolateral preoptic nucleus [204,205]. Again, this output function is influenced by melatonin via its feedback to the SCN. By suppressing neuronal firing through MT_1_-dependent signalling, the wake-related neuronal downstream pathways are inhibited, whereas the sleep-related ones are activated. This represents a major contribution of melatonin to the promotion of sleep initiation, although additional actions of the methoxyindole are also involved (for further details, see [206]).

The control of melatonin formation and secretion is one of the major output functions of the SCN. In terms of rhythm coordination and, on the negative side, chronodisruption, the transmission of light information via the SCN is important to the pineal gland in two aspects. Firstly, the rhythmicity of the SCN, which is entrained by the optic information received from the retina, is imposed on the pineal gland, and thus generates the melatonin rhythm. Although the oscillation of the basically rate-limiting melatonin-synthesizing enzyme, aralkylamine *N*-acetyltransferase (AANAT), is mainly driven by the SCN, rhythmic noradrenergic stimulation finds its counterpart in an endogenous pineal clock, which exhibits cycles in the expression of core oscillator genes [207,208]. In mammals, the function of this pineal clock may be a periodic facilitation of responsiveness rather than the autonomous generation of the melatonin rhythm. Secondly, and of importance with regard to chronodisruption, nocturnal melatonin can be suppressed by LAN [150,209,210,211,212,213,214], an action that occurs in addition to and independent of the phase shifting effects. While resetting depends on the phase response curve and, therefore, varies within the circadian cycle and also in the course of the scotophase, the so-called photic shutoff can take place at any timepoint within the scotophase and depends only on light intensity, duration of light exposure and spectral quality, but not substantially on the circadian phase. This action is particularly pronounced if the spectral composition allows perception by melanopsin, *i.e.*, in the range of 460–480 nm. The photic shutoff causes a rapid cessation of melatonin biosynthesis, and a similarly rapid drop of pineal melatonin concentration and release. Notably, it is observed in animals in which the melatonin rhythm is generated by different mechanisms, either by rhythmic transcription of the *Aanat* gene, as in rodents, or by AANAT phosphorylation and stabilization of pAANAT by a 14-3-3 protein, as in primates and ungulates (details in [199]).

The actions of melatonin are manifold. From a chronobiological point of view, an important effect is the feedback to the SCN [215]. This feedback is the basis for the chronobiotic actions of melatonin, *i.e.*, its ability to reset the circadian clock in the SCN. As with all time cues that reset an oscillator, the extent and direction of the phase shifts depend on stimulus timing, according to a phase response curve (PRC). Although the human PRC for melatonin has been determined [216,217], in practice, the precise PRC of an individual is usually unkwown and varies according to the chronotype and previous illumination schedules. The phase position is, therefore, usually assessed by determining the dim-light melatonin onset (DLMO) [218,219]. The chronobiotic properties of melatonin are the basis for all treatments using the pineal hormone or synthetic melatonergic agonists aimed at readjusting the circadian phases, e.g., after time shifts (jet lag or maladapted shift work) or because of poor entrainment in circadian rhythm sleep disorders (advanced or delayed sleep phase syndromes), in blind people and in circadian-related mood disorders [220,221]. However, it should be emphasized that phase readjustments can be achieved by light exposure. Moreover, combinations of melatonin and light have already been used for this purpose [222,223]. In addition to the feedback to the SCN, melatonin may also entrain or modulate some peripheral circadian clocks, an assumption for which several indications exist [18]. If supported by further data, this will lead to important implications concerning the maintenance of favorable phase relationships between centrally-driven oscillations and autonomous or semi-autonomous peripheral clocks.

In addition to its chronobiotic effects, numerous other actions of melatonin have been described. With regard to its circadian periodicity, they may represent, in respective target cells, the nocturnal up- or downregulation of gene expression, release of humoral factors, neuronal activities and other physiological functions. A detailed description would greatly exceed the scope of this review, but a comprehensive overview can be found in reference [224]. However, it is important to be aware that the complexity of functions is only partially related to the dynamics of the circulating hormone, with its prominent nocturnal peak. Much higher levels of melatonin are found in the tissues of vegetative organs, in particular the gastrointestinal tract, in which the day/night differences are considerably smaller than in the pineal gland or the blood. Among the various extrapineal sites of melatonin formation, immune cells and bone marrow can be specifically mentioned. For detailed information, see reference [224].

A quantitatively important area of melatonin research has been that of antioxidative protection. Again, this is only partially a matter of circadian rhythms and has often been studied under conditions that go far beyond chronobiology, e.g., in fighting sepsis or other forms of high-grade inflammation, in the attenuation of oxidotoxicity by chemicals or insults such as ischemia/reperfusion or brain trauma, and, more recently, in the induction of apoptosis in tumor cells. This interesting field of actions cannot be discussed in detail within the scope of this article. However, it should be briefly mentioned that melatonin displays multiple properties that counteract or even prevent oxidative damage. Apart from direct interactions with reactive oxygen species (ROS) and reactive nitrogen species (RNS), melatonin upregulates several antioxidant enzymes and downregulates inducible and neuronal NO synthases (iNOS, nNOS). While direct radical scavenging requires elevated melatonin concentrations present in some melatonin-synthesizing tissues and, perhaps, in organelles accumulating melatonin as reported for mitochondria [225,226,227], the modulation of gene expression as mediated by MT1 and MT2 receptors can be observed at physiological levels. These effects counteract microglia activation and peroxynitrite formation and support mitochondrial electron flux, thereby reducing electron leakage and thus freeing radical generation, often preventing oxidant-induced apoptosis in nontumor cells [224,227,228,229,230,231]. Without discussing the details of antioxidative protection, emphasis should be placed on an important consequence with regard to chronodisruption and especially to the light-induced melatonin shutoff, as occurs in LAN during shift work. As melatonin exerts numerous antioxidant actions, a suppression of melatonin by LAN should be expected to decrease antioxidative mechanisms inasmuch as they depend on physiological nocturnal melatonin levels [232]. This assumption would be in line with the repeatedly observed increases in lipid peroxidation and decreases in glutathione peroxidase and superoxide dismutase in pinealectomized animals (e.g., [233,234,235,236]). Such a direct reduction of protective capacity must be distinguished from perturbed rhythms in protective enzymes and mitochondrial activity, which are induced by phase-shifting light signals that occur in inappropriate circadian phases and represent another aspect of chronodisruption. However, in the practice of clinical or epidemiological studies, the two undesired changes by LAN, namely dysphased rhythms and melatonin shutoff, have been rarely analyzed.

The effect of light timing on the phase resetting response is also described by a PRC [237,238]. Although comparisons among reported PRCs are difficult to make, due to differences in methodology, they are generally consistent and show that light exposure in the early biological night (from DLMO timing to the minimum core body temperature timing) induces a phase delay of the circadian pacemaker, whereas light exposure in the late biological night/early morning (from minimum core body temperature timing to 8 h later) induces a phase advance. The rest of the day, light exposure does not induce any phase shift, defining the so-called “dead zone”. Most of the PRCs report phase delays of less than 12 h (type 1, or weak, *versus* 0 or strong, according to the average slope of the plot of the initial and final circadian phase following a resetting stimulus) [239,240,241,242]. Other studies [243,244] showed type 0 resetting and accompanying amplitude suppression following three consecutive days with light pulses. While most of light PRC studies have been performed with white light, a recent study evaluated the PRC obtained under blue light exposure (480 nm), reporting similar results [245].

Chronodisruption, including melatonin shutoff, has been assumed to play an important role in a variety of health problems. However, it is necessary to distinguish among the different disorders or diseases and their mechanistic causes, and among the methods used for demonstrating the relationship to chronodisruption. This becomes particularly evident in the numerous epidemiologic studies, in which health problems related to shift work have been evaluated. Epidemiology is frequently confronted with the general problem of heterogeneity, and this is very much the case in shift work [246]. This not only concerns differences in lifestyle habits and nutrition and the type of work (including exposure to other unhealthy factors), but also the various forms of shift work (e.g., length and direction of rotating shifts), the duration of employment under shift schedules, and periods after the end of shift work. This latter point is relevant insofar as health problems progressively emerge with age and may become evident only after the periods of shift work have already ended.

Despite these methodological difficulties, the association of shift work with health problems has been demonstrated in a number of studies. This is most evident in the complex of metabolic syndrome, cardiovascular diseases (especially coronary heart disease [247,248,249,250,251,252]), and diabetes type 2 [253,254,255,256]. The mere demonstration of such associations is, however, not entirely sufficient. It is also important to know whether shift work induces health problems or aggravates disorders. This latter aspect has been addressed in a study by Lin *et al*. (2009) [257], in which a pre-existing metabolic syndrome was shown to be aggravated by shift work. Another point concerns the changes in eating habits induced by LAN or work at night. In fact, altered food intake and obesity were shown to be induced by shift work and to be associated with elevated blood pressure [258,259,260,261]. These changes were even observed under conditions of a fixed shift work schedule [260]. Therefore, the health problems arising from shift work and LAN might be assumed to be indirectly caused by an altered food intake. Another indirect influence may arise from sleep disturbances. Sleep deficits and interruptions are also known to be associated with changes in eating behavior and obesity [262,263,264,265]. Of course, an increase in body mass index related to eating at night also entails the aspect of circadian rhythmicity in nutrient uptake and metabolism, but the mechanistic relationships are not that simple, because of the demonstrable association between sleep debt and obesity. Nevertheless, although food intake and metabolic utilization are influenced by sleep loss, a recent study has shed light on the importance of circadian misalignment on insulin resistance. Authors showed that insulin resistance is promoted by circadian perturbance, under conditions of controlled sleep loss [254]. Although sleep restriction also promoted insulin resistance, the effect was considerably higher under circadian misalignment. Importantly, the change in insulin sensitivity was associated with increases in inflammatory markers, a finding that is also relevant to numerous other diseases and merits further investigation on a broader scale. Moreover, it is indicative of a considerable influence of chronodisruption beyond rather simple relationships to the amount of food and changes in body mass. It would be of interest to elucidate the role of melatonin deficits in this context.

With regard to both melatonin and other hormones or humoral factors that undergo circadian cycling, more information is required concerning LAN-induced metabolic changes. Evidence is currently accumulating that shift work alters the plasma levels of resistin, ghrelin, leptin and adiponectin [247]. These findings are not only of interest with regard to the regulation of food intake and nutrient metabolism, but also in terms of inflammation and atherosclerosis. In particular, the leukocyte-derived factor resistin has been discussed as a mediator of cardiovascular risk in rotating shift work [266]. Again, the question remains to what extent circadian misalignment is decisive and the potential contribution of melatonin shutoff by LAN.

It should be briefly mentioned that LAN is not only a matter of shift work, rather it must be considered as a contributing factor in gerontological problems. This has been recently addressed in a study of an elderly population [267]. Moreover, an association between midlife insomnia and mortality has been reported [268], which may also be of relevance to aged subjects.

As mentioned above, melatonin is known to be a potent antioxidant. One of the predictable consequences of a nocturnal melatonin shutoff by LAN is, therefore, an increase in oxidative damage to biomolecules. In addition, circadian perturbations by mutations in clock genes or repeated phase shifts have also been shown to increase oxidative damage [269] and to reduce lifespan in animals [270,271]. In light of these relationships, it is surprising to observe that the connection between shift work and oxidants has been rarely studied. Two investigations have demonstrated increases in 8-hydroxydeoxyguanosine in the DNA of shift workers [272,273]. In the future, this aspect should be more extensively investigated, and also with regard to its consequences in numerous other diseases. Moreover, it will be necessary to clarify the contribution of inflammatory responses to LAN-induced damage in biomolecules.

Another area in which inflammation and mutations induced by oxidative stress are of particular importance is cancer. The possible association between shift work and cancer has been vividly discussed during the last years, especially after a respective classification by the International Agency for Research on Cancer in 2007 (*cf.* [274]). However, this relationship to cancer is not nearly as clear as initially thought. In various types of cancer that had been suspected to be promoted by shift work, epidemiology failed to support this assumption. The best documented association with shift work concerns breast cancer, but despite a remarkable degree of variability among studies and a final conclusion that this relationship is demonstrable, it fails to represent a major risk factor [275,276,277,278,279,280,281,282,283]. Other cancers with a high likelihood of being convincingly related to shift work are colorectal cancer [72,284], ovarian cancer [285] and non-Hodgkin lymphoma [286,287]. Apart from the general problems related to the heterogeneity of epidemiology, a major problem of the respective cancer studies is the lack of a mechanistic explanation. Disturbed or misaligned circadian rhythmicity is frequently mentioned, but the reasons for the promotion of cancer have remained unclear. Moreover, the necessity of distinguishing between perturbed/shifted circadian rhythms and melatonin shutdown has not frequently been seen. On the other hand, both chronobiology and melatonin physiology offer manifold possible nexuses to cancer, such as mutations in clock genes that make an organism cancer-prone, cancer-related alterations of melatonergic signalling (summarized in [18]), and proinflammatory effects of melatonin [28,288]. Additional hints can be obtained from preclinical studies in animal models. For instance, the finding that LAN favors resistance to an anticancer drug, tamoxifen [289], must have a mechanistic explanation that might be relevant to the development of cancer in humans. Additional animal data from experimentally well-controlled studies showing that chronodisruption promotes tumorigenesis, along with reductions in life span [290], may be more convincing to researchers of the connections between cancer and disturbed circadian and melatonin physiology than poorly controlled epidemiological studies affected by many confounding factors. Although the relevance in humans must be ultimately demonstrated, the mechanistic elucidation may be easier in animals.

A further future demand has to be the monitoring of chronodisruption in humans. To a certain extent, this may be done by ambulatory circadian monitoring (ACM), on the basis of wrist actigraphy, thermometry or body position measurements, for example [291,292]. However, all such data must be interpreted with due caution, since chronodisruption not only leads to changes in the oscillators, it also has negative and positive masking effects, which must be identified and, to the extent possible, removed to yield a purified time pattern. With regard to melatonin, determinations of dim light melatonin onset (DLMO) have been applied in sleep research, especially concerning circadian rhythm sleep disorders [219], and most recently using a DLMO “hockey-stick” variant [218], and have been compared to ACM data [292]. Although this can also provide valuable data on circadian changes, the other aspect of melatonin shutoff is not sufficiently accessible using this method. If the loss of melatonin by LAN should turn out to be more important than circadian perturbations, the only way to monitor it in a meaningful manner would be through the determination of melatonin. This can be most easily done using salivary melatonin, but it requires the exclusion of confounding factors, such as melatonin-containing food and beverages, especially coffee.

## 6. Impaired Retinal Light Input

There are two situations in which the input pathways can be damaged: aging, since the crystalline becomes more opaque and thus the light finds it more difficult to enter the circadian system, and in visual pathologies, in which, depending on the injury, circadian photoreception may or may not be possible.

### 6.1. Aging

The human circadian system, like other cell, organ or systems in the organism, undergoes processes of maturation and aging. In the circadian system, profound alterations occur at all levels, from the inputs to the outputs and in the circadian clock itself [293,294].

**Figure 7:**
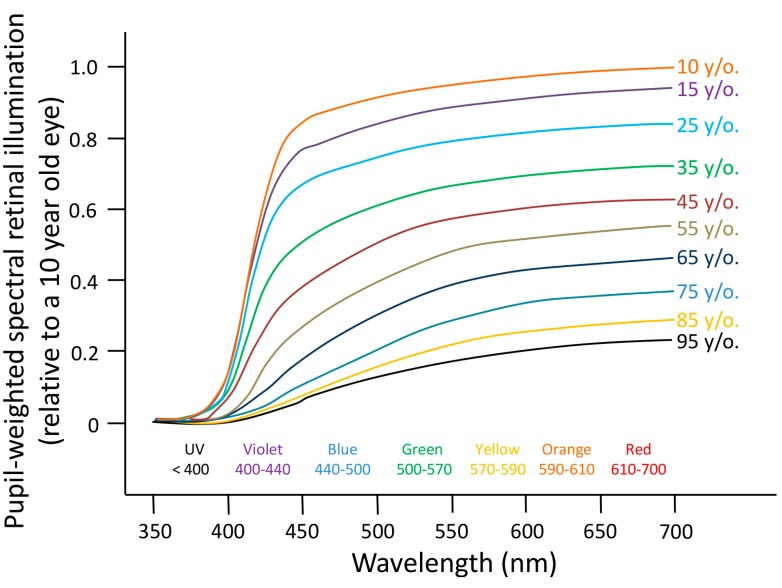
Age-related losses in retinal illumination due to decreasing crystalline lens light transmission and pupil area. The percentage of loss per decade is reasonably uniform and most prominent at shorter violet (400–440 nm) and blue (440–500 nm) wavelengths (reproduced from [294]).

Regarding the input pathways, there are some structural and functional changes. Thus, as absorption in the crystalline lens for shorter visible wavelengths (400–500 nm) [295] increases substantially with age, at the same time that the pupil diameter tends to decrease (miosis), the effective retinal exposure received under the same ambient lighting conditions is lower in the aged, as compared to the young, eye [296,297]. Very elderly individuals retain just 10% of the photoreception of a 10-year-old, and therefore would require ten times brighter exposures from identical light sources to maintain youthful circadian performance [294]. Figure 7 shows the decline in circadian photoreception over the decades and its improvement following cataract surgery, implanting various intraocular lenses (IOLs).

The possible effects of these changes on the circadian rhythms are not clear. Both opacity and miosis would produce progressive age-related losses in circadian photoreception, in terms of phase shifts and melatonin suppression [294,298]. However, other authors [295] reported no changes in melatonin suppression and phase shift under short wavelengths of light between elderly and young subjects, despite reporting decreases in transmittance and pupil diameter. This would be in accordance with a recent study by Herbst *et al*. (2012) [299], in which an enhancement of the pupillary light reflex mediated by ipRGCs in older subjects with high lens opacity was found. These findings would suggest the existence of compensatory mechanisms that allow the aged eye to transmit non-visual light information with the same efficiency.

Concerning the effects of aging on retinal cells integrity, rods and retinal ganglion cell populations seem to decline with age, but cone photoreceptor populations appear to remain relatively stable [300,301]. The effect of aging on ipRGC is still not clear, although in glaucoma, which is associated with ganglion cell loss, ipRGCs are resistant to ocular hypertension (at least in rodents) [302].

With age, the biochemistry and morphology of the suprachiasmatic nuclei is progressively altered. Although normal aging does not decrease cell size or number in the master pacemaker [303], and although the SCN is relatively resistant to neurodegeneration by excitotoxic insults [304], alterations in peptide expression (vasoactive intestinal polypeptide, VIP; and arginine vasopressin, AVP) and a reduction in the amplitude of the circadian rhythms of electrical activity have been described [305]. Senescence also impairs the molecular clock. Thus, the expression of *Clock* and *Bmal1*, but not *Per1* and *Per2* genes is altered in the SCN of rodents [306,307,308]. It is likely that telomere shortening, reduced activity of the transcription factor CREB and changes in the MAP kinase cascade, which accompany cellular senescence, are responsible for the attenuated expression of circadian clock genes [309]. The impaired expression/activity of important circadian core oscillator proteins, such as BMAL1 and CLOCK, in turn, further contributes to the development of age-related pathologies [310].

Regarding overt rhythms, in general terms, aging is characterized by phase advance, fragmentation, amplitude dampening and period shortening of the rhythms [293]. However, there is no complete agreement regarding the latter, since period length has been found to be similar to that of young people in a forced desynchrony protocol affecting melatonin and core body temperature [311]. In addition, the main output, melatonin, is dampened by pinealocyte receptor changes and, sometimes, pineal calcification and size reduction [293,312,313]. Still there are authors who report that the melatonin rhythm amplitude is maintained in healthy elderly subjects [314]. It should be noted that the changes in circadian patterns can be considerably aggravated in neurodegenerative disorders, in particular, in Alzheimer’s disease, in which the rhythms break up into different components. These changes have been explained by SCN degeneration [315]. The nature of this degeneration, whether caused primarily by loss of connectivity or neuronal function and numbers, has not been sufficiently clarified. However, the impairments are also evident in the reductions and decomposition of melatonin levels and nocturnal patterns, respectively [316].

In addition, the sensitivity and exposure to the major *zeitgeber*, the light-dark cycle, is also altered on a civilizational basis, and even more so during aging [293,312,313]. In most individuals, exposure to sunlight is slowly and progressively reduced with age [294]. In industrialized countries, young adults typically receive only 20–120 min of daily light exceeding 1000 lux [48,314,317,318], while institutionalized elderly subjects receive only 1/3–2/3 of this daily bright light exposure [319], even in nursing homes, where in some cases, they are exposed to low light intensities during the day for many years (reviewed in [320]).

### 6.2. Blindness

As previously mentioned, ocular light exposure is the most important environmental circadian synchronizer. So, how are the circadian rhythms affected in visually impaired subjects? For some time, abnormal hormonal patterns have been reported in some visually impaired patients [321]. In 1940, Remler studied the rhythms in rectal temperature, heart rate, blood pressure and urinary excretion in blind subjects, obtaining normal 24-h rhythms in some of them and inverted rhythms in others [322]. Other authors have found abnormalities in the secretion profiles of corticosteroids in the majority of blind subjects studied [323,324,325,326]. However, it has been demonstrated that some blind subjects with no conscious perception of light (NPL) present normal 17-hydroxycorticosteroid (17-OHCS) [323] and 11-hydroxycorticosteroid (11-OHCS) secretion patterns [324], although most of them presented abnormal rhythms for these hormones and cortisol [325,326]. Other studies also failed to find any differences in the circadian phase of plasma and urinary excretion of 17-OHCS [327], cortisol [328] and norepinephrine and epinephrine [329] between sighted subjects and blind subjects with NPL.

In 1983, Lewy and Newsome [330] found that 6 out of 10 blind subjects presented abnormally timed melatonin rhythms. Later on, Sack and colleagues carried out a more extensive longitudinal study of the plasma melatonin rhythms in 20 NPL subjects, confirming a heterogeneous distribution of melatonin rhythm types (15% normally phased, 15% abnormally entrained, 55% free-running with periods from 23.86 to 25.08 h and 15% arrhythmic) [331]. Other authors directly studied the possible association between loss of light perception and sleep disorders [332] and the rhythm of 6-sulphatoxymelatonin (aMT6s) [333]. Sleep disturbance was recorded in nearly 50% of the blind subjects. However, the prevalence was higher (66%) and the sleep disturbance was more severe in the NPL group, as compared to blind subjects with a visual acuity of LP or better and to sighted controls [332]. Skene *et al*. [333] also found a higher incidence of aMT6s rhythm entrainment abnormalities (or free running) in the NPL group (76%) than in the LP group (23%), finding, however, a certain percentage (29%) of NPL subjects with normally entrained aMT6s.

Another prediction that can be made considering the importance of light for circadian entrainment is that sleep disorders would be more prevalent in blind as compared to sighted subjects, or in those with NPL, as compared to those with some degree of LP. Miles and Wilson reported that 76% of blind subjects with a range of visual loss complained of a sleep-wake disorder with cyclic or episodic symptoms, which is an important characteristic of circadian rhythm sleep disorders [334]. These same authors showed that a blind man with NPL had non-entrained “free-running” sleep-wake, and other rhythms when the subject lived freely without restriction [335]. These abnormalities persisted despite attempts to entrain the rhythms using a strict regime of bedtime, meals, and activity, or knowledge of clock time. In 1990, Martens et al reported that 71% of NPL subjects (*n* = 16) complained of a chronic sleep disorder associated with increased sleep episodes and increased daytime sleep [336].

**Figure 8:**
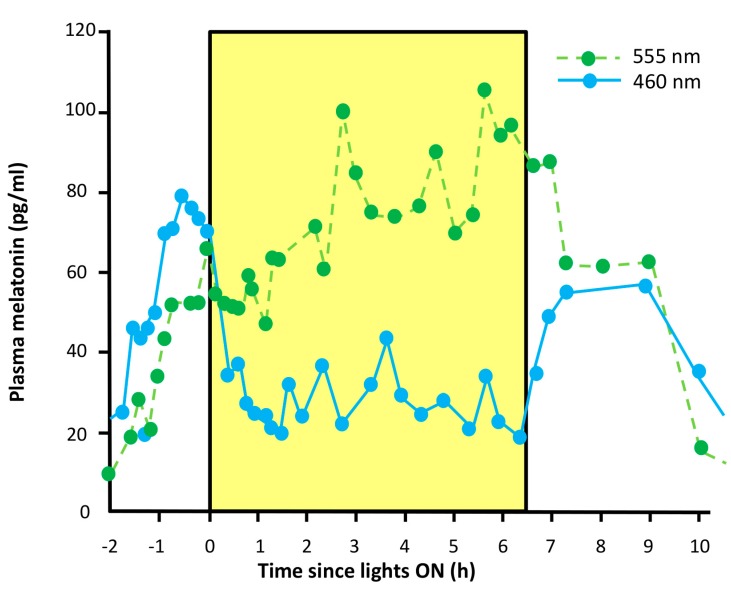
Short wavelength light sensitivity for melatonin suppression. Comparison of the effects of 460 nm (blue circles) and 555 nm (green circles) light exposure on melatonin suppression in a blind man. The graph represents the direct effects for melatonin suppression of exposure to green (555 nm) and blue (460 nm) monochromatic light on the male subject. Exposure to 555 nm light caused no suppression of melatonin as compared to the corresponding clock time the previous day, whereas exposure to 460 nm light suppressed melatonin and maintained the suppression effect throughout the entire 6.5 h exposure (reproduced from [140]).

All these studies demonstrate the relationship between certain visual pathologies and circadian rhythm disorders. However, disorders of the visual system do not always hamper the circadian effects of light, thus demonstrating a functional separation of the visual and circadian photoreception pathways. Thus, the majority of legally blind people who preserve some degree of LP, even with very little usable vision, have normally entrained circadian rhythms [337]. Moreover, it has been demonstrated that some blind people with NPL retain normal circadian phase-shifting and melatonin suppression in response to blue light (Figure 8), even in the absence of any functional rods or cones, as assessed by conscious ability to detect light, visually-evoked potentials, or an electroretinogram [122,139]. As expected, if their eyes contain fully functional circadian photoreceptors (ipRGCs), these individuals exhibit normally entrained 24 h rhythms under real-world conditions and do not report sleep disorders [122].

Thus, we conclude that visual pathologies can be divided into two groups with respect to their effects on the circadian system: visual pathologies compatible with circadian photoreception (PCCP): those visual pathologies in which ipRGCs and all the nerve pathways from the retina to the SCN are still functional; visual pathologies incompatible with circadian photoreception (PICP): those in which ipRGCs or the optical nerve are not functional in either eye.

Therefore, the treatment for both types of visual pathologies in relation to the associated circadian disorders would be different. In the first group (PCCP), light therapy alone (enhancing the contrast between day and night) may be sufficient to entrain the circadian rhythms in these blind people. It is important to mention that some totally blind people are not properly exposed to light (since they cannot use it to see), so even if they retain circadian photoreception, their circadian system may not be entrained. In the second case (PICP), the treatment would be mainly pharmacologic, through the administration of melatonin at the appropriate dose and timing [101,338,339,340,341].

But how can we assess the functionality of the circadian photoreception nerve pathways? There are some procedures to demonstrate the persistence of circadian photoreception in blind people:

(a) Pupillary light reflex. Blind people with PCCP will retain the pupillary light reflex under short wavelength light stimulus.
(b) Melatonin suppression. Totally blind people suffering from a PCCP will respond to a pulse of light with the suppression of melatonin production.

## 7. Circadian Healthy Light

Scheduled bright light exposure is an effective countermeasure for sleepiness and fatigue in shift workers and those suffering from jet lag or delayed and advanced sleep phase syndrome. Moreover, the beneficial effects of light on mood, sleep quality, and/or cognitive performance have been found in quite different pathological conditions, including Parkinson’s and Alzheimer’s diseases.

Although circadian entrainment in humans can be attained with lower light intensities [342], lighting levels of at least 1000 lux at eye level have been proposed to be necessary [343,344]; however, this level of lighting is not available in most offices and industrial areas [343,344]. Working indoors during the day implies light intensities of 40–200 times lower than being outdoors. Since the maximal human circadian spectral sensitivity occurs at 460–480 nm, diurnal lighting should not be poor in this part of the spectrum [116,345]. Thus, all the evidence indicates that daytime lighting should not have a low percentage of blue wavelengths and should present greater intensities than those that are usually found. However, not only intensity and spectrum are important in order to obtain healthy day lighting; glare and spatial distribution also need to be considered. The use of devices capable of modulating light intensity throughout the day in a way that mimics the Sun has also been recently proposed [346].

As reviewed earlier, light at night can have negative effects on circadian rhythms and on health in general [116], especially if it is enriched with wavelengths from 460 to 480 nm [347]. Thus, the luminous energy associated with light radiation, especially that from the short wavelength part of the visible spectrum (400–500 nm), can cause toxic effects in the eye [348]. Short wavelength light can penetrate the cells and their organelles, inducing the generation of reactive oxygen species (ROS) in retinal pigment epithelium mitochondria and even apoptosis, potentially caused by ROS-damaged mitochondrial DNA, as reported in *in vitro* studies [349,350]. A recent study *in vivo* has also demonstrated that white LEDs (with high content in blue light) at domestic lighting levels cause retinal injury in a rat model [351], although it should be noted that the animal model used is nocturnal and albino, and thus any extrapolation to humans would be inaccurate. On the other hand, it also could be argued that natural sunlight contains greater blue light intensity. However, it should be noted that retinal physiology changes between day and night (see Section 4.1.2), so it could be hypothesized that blue light at night could entail a greater risk to the retina integrity. Thus, nocturnal lighting should avoid these specifically active wavelengths [352,353,354] due to their negative effects on both the circadian system and the eyes themselves. Technically, spectrum modifications can be achieved through two procedures: (a) by filtering the 460–480 band of the spectrum or (b) by modulating the intensity and spectrum, depending on the time of the day, using light generated by independent red, green and blue LEDs sources, for example.

Light has been also used as a therapy to treat several disorders, including seasonal affective disorders (SAD) and disrupted sleep-wake rhythms [355,356,357]. The idea is to increase the *zeitgeber* strength by enhancing the light exposure during the day, and thus the contrast between the day and the night. The most critical phases for circadian light effects are found after dusk or before dawn, so if exposure to artificial light of sufficient intensity occurs at these moments, it will cause phase shifts (a delay or advance) [358], contributing to the entrainment of the circadian pacemaker to the 24-h light-dark cycle. Recently, a variation of this therapy has been developed, consisting of simulating dawn and dusk (Dawn-Dusk Simulation, DDS). Since the early pioneering times of Chronobiology, twilight has been known to influence the entrainment of circadian rhythms [359], in particular, enhancing the coupling to a weak *zeitgeber* [360]. DDS is based on imitating the outdoor twilight and sunrise transitions. With this treatment, a gradual onset of dusk and dawn is adjusted to the patient’s sleep time. DDS can be regarded as a “natural” light therapy because of its lower and gradual changes in light intensity. DDS has been shown to be a successful therapy in the treatment of some psychiatric disorders [356,361] and patients who suffer circadian sleep-wake cycle disorders [356,362]. Moreover, it is known that DDS induces small advances in the circadian rest-activity rhythm by triggering an earlier onset of the most restful period of the night [356].

It is clear that although we can design some strategies to create a “melatonin-friendly light”, there would probably be some cases in which protecting the melatonin rhythm would entail extra problems. It is important to be aware that the melatonin rhythm is also affected by parameters other than cycling light intensity. As already mentioned in the section on aging, impaired function of the SCN or of signal transmission to the pineal gland can reduce nocturnal melatonin secretion during senescence and, to an even greater extent, in patients with Alzheimerʼs disease (AD) and other forms of dementia [316,363,364]. Although the causes of dysfunction are not necessarily or primarily a matter of light perception, light therapies have been tested in AD patients, (for examples, see references [316,363,364,365,366,367,368]). In general, the improvements reported in these studies were relatively modest. Although changes in the proportion of daytime/nighttime activities were observed, in addition to some behavioral benefits [365,369], improvements of sleep and circadian rhythmicity usually remained marginal. The efficacy of bright light therapy on sleep consolidation and effects on the circadian system depended on the progression of the disease [366,370]. While improvements were demonstrable in earlier stages, this was decreased in the case of advanced AD. At the melatonin rhythm level, light therapy remained relatively inefficient in late AD. In particular, reductions of daytime melatonin were not achieved, contrary to findings in patients with other psychiatric disorders [363]. These observations are supported by more recent data, which indicate, however, a relatively early onset of SCN and pineal dysfunctions [371]. Reductions in pineal melatonin secretion, as well as disrupted clock gene oscillations in the pineal, were already demonstrable in Braak stages I–II [371]. The idea that a combination of bright light therapy with melatonin administration may be helpful, which has received some clinical support [372], may be critically viewed with regard to extreme reductions in the expression of melatonin receptor MT1 in the SCN of AD patients [371]. Nevertheless, interindividual differences may exist, and melatonin may be beneficial in SCN-independent or only partially dependent functions. Therefore, the use of melatonin should not be precociously ruled out, in the absence of additional studies that consider these possibilities.

Reduced melatonin levels have been also observed in various other diseases and disorders (summarized in [364,373]). These include various cases in which there is no reason to assume dysfunction of the SCN. In particular, such reductions have been found to accompany several stressful or painful conditions, such as Menièreʼs disease [374], fibromyalgia and neuralgia [375], migraines [376,377], heart diseases [378,379,380,381,382,383,384], critical illness [109,385,386] and cases of cancer [387,388], in which the contribution of stress and pain has remained unclear, as well as in some metabolic diseases, such as acute intermittent porphyria [389,390], and notably, diabetes type 2 [391,392]. In some neurological disorders, decreases in melatonin are only observed in subpopulations of affected individuals or in a very limited number of subjects studied [364]. As damage to the SCN does not seem to be causal to the reduced melatonin levels, one might be inclined to assume that increases in *zeitgeber* strength (e.g., higher light intensities or an enhanced proportion of blue light) might be able to correct the circadian deficits affecting the melatonin rhythm. This may be possible in less severely affected patients, in which the melatonin rhythm would return to normal anyway after the end of the stress- or painful conditions. However, this has not been sufficiently studied. In the case of critical illness in which the patient receives intensive care, attempts at correcting the melatonin level by a light-dark cycle have been unsuccessful [109]. Some skepticism may be also due in advanced diabetes type 2, at least as far as the disease has already led to a neuropathy (*cf.* ref. [391]). With regard to the more recently discovered connection between diabetes type 2 and AD in terms of insulin resistance in both vegetative organs and the brain [393,394,395,396,397], it is still unclear the extent to which resistance to insulin may already affect neuronal functions in patients who have an otherwise asymptomatic neurological condition.

With regard to pathological deviations, the usefulness of strategies to support the melatonin rhythm must be judged according to a different barometer. In all disorders in which circadian malfunction or poor entrainment are causal, *i.e.*, in circadian rhythm sleep disorders and circadian-related forms of depression, such as borderline personality disorder (BP) or SAD; enhancements of *zeitgeber* strength by means of bright light, more intense blue light and, eventually, twilight phases around dawn and dusk are promising. Additional medication with melatonin or synthetic melatonergic drugs in the evening may further enhance success. In other diseases discussed in the previous paragraph, the chances of improvement are either limited, low or have not been tested.

Difficulties in readjusting or restoring the melatonin rhythm are of an entirely different nature in the case of shift work. Although it should be possible to strongly reduce the blue and green fraction of the light spectrum without too great of a reduction in overall light intensity, the question remains to what extent the working capacity of an individual is affected by a light quality that only moderately reduces melatonin [398]. The maintenance of a high nocturnal melatonin level may well reduce the alertness of a worker and cause undesired safety problems. This has to be weighed against more convenient durations and sequences of shift periods.

As already mentioned, in the developed countries, the usage of smartphones, tablets and other electronic devices has been widespread over the last few years. Some applications have been recently developed to reduce the negative effects of their use at night. In general, they work by adjusting the display color temperature according to the natural light-dark cycle. Thus, they avoid high color temperatures after sunset, while permitting them during the day.

## 8. Conclusions

- Humans have altered the natural light-dark cycle contrast by increasing light at night and spending most of their time indoors, with low light intensity exposure during the daytime.
- In order to maintain the health of our circadian system, appropriate lighting levels during the day should be recommended, even in certain cases of blindness (always under the supervision of an ophthalmologist).
- In addition, diurnal lighting should not be poor in wavelengths in the 460–480 nm range, since maximum human circadian spectral sensitivity occurs in this part of the spectrum.
- On the other hand, darkness during the night is desirable, and when illumination is a must, the abovementioned specifically active wavelengths should be avoided, shifting to a more reddish spectrum. Interestingly, bluish wavelengths are the ones that most interfere with astronomical observations, and “whiter” light is likely to increase the potential range of environmental impacts on other living organisms. Thus, reducing light pollution would have positive effects, not only on human health, but also in terms of cultural and environmental aspects.
- Since our recently acquired lifestyle habits seem to require illumination at night, new lighting technologies using the favorable spectrum and intensity should be developed to preserve circadian system functioning both at night and during the day inside buildings.
